# A questionnaire study on the knowledge, attitudes, and practices of fluid replacement and urination among Chinese elite athletes

**DOI:** 10.1371/journal.pone.0275685

**Published:** 2022-10-12

**Authors:** Ge Song, Yi Yan, Haotian Zhao, Junying Chen, Yimin Deng, Wenge Zhu, Lingyu Sun, Guansheng Ma

**Affiliations:** 1 Department of Sport Biochemistry, School of Sport Science, Beijing Sport University, Beijing, China; 2 Jiangnan University, Wuxi, China; 3 Department of Nutrition and Food Hygiene, School of Public Health, Peking University, Beijing, China; 4 Laboratory of Toxicological Research and Risk Assessment for Food Safety, Peking University, Beijing, China; University Medical Center Utrecht, NETHERLANDS

## Abstract

**Objective:**

To evaluate the knowledge, attitudes and practices (KAP) of Chinese elite athletes about fluid replacement and urination.

**Methods:**

A cross-section study was carried out among Chinese national and national youth teams from March to April 2020, using a pretested questionnaire. The 42-questions questionnaire was designed to assess the KAP regarding fluid replacement and urination. The questionnaire included knowledge of fluid replacement (KFR), attitudes of fluid replacement (AFR), knowledge of urination (KU), and attitudes of urination (AU), which were awarded 20 scoring points. Descriptive statistics, independent samples t-tests, one-way ANOVA, Pearson’s correlation analysis, Multiple linear stepwise regression and Chi-square test were performed.

**Results:**

A total of 779 valid questionnaires were collected and the effective rate is 98.4%. We finally conducted an assessment of 646 questionnaires of elite athletes. The mean score for KFR, AFR, KU, and AU was 2.8±1.3, 2.3±0.6, 3.0±1.5, and 2.1±0.8, respectively, with higher scores indicating positive hydration knowledge and attitudes. KFR and AFR scores of winter sports athletes were higher than those of summer sports athletes(*P*<0.05). Athletes who had lower athletic grades and training years had a worse KFR(*P*<0.05). Only 31.0% athletes knew that rehydration should be carried out before, during, and after training, which was scarcer among women, lower-athletic grades athletes, or athletes with lower training years (*P*<0.05). Male athletes had a worse KU but a better AU than female athletes(*P*<0.05). And athletes who were international-class athletic grades had the highest KU scores(*P*<0.05). The athletic grades and sport events were the main factors influencing the total scores of knowledge and attitudes (*P*<0.05, 95% CI -0.789–-0.168,95% CI 0.025–1.040). Most of athletes tend to get hydration knowledge from internet. In practices, thirst is the main reason for rehydration (77.9%). The percentages of athletes with normal urine color (42.0%), frequency (75.0%,) and volume (20.0%) were low.

**Conclusions:**

These findings indicate that Chinese elite athletes did not have sufficient KAP on fluid replacement and urination, more marked in the individuals who were summer sport events, the lower athletic grades and in lower training years. It is recommended that education should be provided in the early stages of professional training for athletes.

## Introduction

As we know, maintaining fluid balance is critically important for sustaining athletic performance [1–4], especially for elite athletes [5–7]. Despite this, most athletes experience deterioration in hydration state during training and competition, which has an inevitably negative impact on performance. Arnaouts and his colleagues [8] have reported that the prevalence of hypohydration among elite young athletes is very high based on urine specific gravity (USG) and urine color. Thus, there are many studies focused on pursuing strategies that can assess and sustain the hydration state [9–13]. Because athletes are the main enablers of these strategies, their knowledge, attitudes and practices (KAP) on achieving and sustaining an optimal hydration state is the basic guarantee to ensure the effective implementation of strategies [14,15]. In order to create awareness of athlete, insight into the gaps of KAP regarding fluid replacement and urination due to exercise is important, which will help improve the pertinence and effectiveness of education.

In China, there is no study to evaluate the athlete’s KAP of fluid intake and urination, which is imperative to conduct studies to be helpful for improving the performance of China at the 2020 and 2022 Olympic games. Therefore, the objective of this study was to discover the KAP characteristics of the Chinese elite athletes regarding fluid replacement and urination, and evaluate the associated factors affecting the KAP. The outcome of the study will provide a scientific basis for further development of health education and targeted rehydration strategies among Chinese athletes.

## Materials and methods

### Study design, participants and data collection

This cross-sectional study administered an anonymous questionnaire survey using Wen-Juan-Xing online platform in China [16]. The survey was conducted among Chinese national and national youth teams from March to April 2020. The median temperature during the day in that period was 15±5°C. The study was conducted under the Declaration of Helsinki and was approved by the ethical review committee of Peking University (IRB00001052-19051).

A simple random sampling formula N = t^2^P(1-P)/e^2^ was used to determine the sample size [17]. When N value is the sample size, the t value is 1.96 corresponding a 95% confidence level, P value is expected prevalence of the exercise population who recognize the importance of fluid replacement for health, which was estimated at 34.2% according to relevant literature and similar surveys [18], and e is the precision level of 4%. The data was calculated with a sample size of 540 subjects. An electronic link was sent to the athletes by the team doctors and coaches of Chinese national and national youth teams through WeChat. The questionnaire was in Chinese. The English translation version of the questionnaire has been uploaded as a supplementary file. Then we recruited healthy athletes based on the medical examinations provided by team doctors. To obtain written informed consent from those who indicated a willingness to participate, nutrition experts described the objectives and procedures of the study to facility managers and athletic center staff, including team doctors. Informed consent was obtained from each participant before they participated in the study.

Elite athletes are the athletes who compete for the highest national championships such as the Olympic Games [19], and excludes physical education in school or ‘open-level’ sports teams [20]. According to the Athletic Grade Standards of China, from the highest competitive ability to the lowest, all the athletes were divided into 5 grads: international-class athlete (IA), national-class athlete (NA), first-class athlete (FA), second-class athlete (SA) and third-class athlete (TA). IA, NA and FA athletes who belongs to elite athletes were analyzed in this study.

Summer sports were defined as the events included in the Summer Olympic Games, track and field, swimming, archery, boxing, trampoline, cycling, fencing, sailing, shooting, weightlifting, wrestling, etc. were included in this study. And winter sports were defined as the events included in the Winter Olympic Games, figure skating, freestyle skiing, ski jumping, cross country skiing, ice hockey, curling, speed skating, short track, biathlon, etc. were included in this study.

### Questionnaire design

The questionnaire was slightly modified from CHI’s questionnaire [18]. The adaptation was completed mainly in sports nutrition area. In order to ensure the effectiveness of the questionnaire design, we set up a unified survey description at the beginning of the questionnaire including research objectives, research methods and matters for attention in filling. Then we conducted a small survey on 20 athletes using the adapted questionnaire as a pilot study. After that, the questionnaire was revised again based on the issues found in the pilot study. Later, this questionnaire was reviewed and approved by five experts in the professional area for the context validity. Finally, we completed our questionnaire, and upload the final version into the WEN-JUAN-XING online platform for the online survey.

Overall, there were 42 questions in the questionnaire consisting of 7 parts: the demographic information (8 questions), knowledge of fluid replacement(KFR, 7 questions), attitudes of fluid replacement(AFR, 5 questions), practices of fluid replacement(PFR, 4 questions), knowledge of urination(KU, 6 questions), attitudes of urination(AU, 6 questions), and practices of urination(PU, 6 questions). Participants assessed urine color with a simplified pictorial color scale [21,22].

### Statistical analysis

All data were exported from the platform into EXCEL format, and SPSS Statistics 22.0 was used for data analysis. One point for each correct answer of KFR or KU, and one point for each positive related attitude. The maximum score of KFR was 7, and the maximum score of AFR was 3. The maximum score of KU was 6, and the maximum score of AU was 4. A score of 4 or higher is considered adequate knowledge. The maximum total score of knowledge and attitudes of fluid replacement (TFR) and the maximum total score of knowledge and attitudes of urination (TU) were both 10.

Independent T-tests and one-way ANOVA were used to investigate any differences among groups for the scores of knowledge and attitudes. Multiple linear stepwise regression was used to predict the influence factors of the total scores of knowledges and attitudes (S1 Table in S1 File). Correlations between the total scores of knowledge and attitude on fluid replacement and urination behavior were determined using Pearson’s Correlation coefficient. The Chi-square test was used to identify any differences among groups for fluid replacement and urination. Bonferroni correction was used for pairwise comparison. *P* < 0.05 was considered as statistically significant.

## Results

### Characteristics of participants

In general, 792 eligible respondents participated in the survey. After taking the completion of all the questions in this study as the standard for validity and excluding the unqualified questionnaires (incomplete filling, not filling as required or <100 s to complete the questionnaire), the qualified questionnaire rate was 98.4% (779 out of 792 questionnaires). 664 questionnaires of IA, NA, and FA were analysed. The average age of all the respondents was 22 years, and the average training years was 8.5 years (Table 1).

**Table 1:** Demographic characteristics of participants.

Demographic group	Classification	Age	Frequency (n)	Percentage (%)
Total		22±4	646	100.0
Sport events				
*S*	Summer sports	22±4	491	76.0
*W*	Winter sports	20±4	155	24.0
Gender				
*M*	Male	22±4	275	42.6
*F*	Female	21±4	371	57.4
Athletic grades				
*IA*	International-class athlete	24±4	137	21.2
*NA*	National-class athlete	22±4	328	50.8
*FA*	First-class athlete	19±3	181	28.0
Training years				
*Q*1	N≤3	17±2	93	14.4
*Q*2	3<N≤ 6	20±3	129	20.0
*Q*3	6<N≤9	22±2	155	24.0
*Q*4	9<N≤12	23±3	163	25.2
*Q*5	N>12	27±5	106	16.4

### Knowledge and attitudes of Chinese elite athletes

#### Mean scores of KFR, AFR, and TFR

As shown in Table 2, there were significant differences in the scores of KFR among sport events, athletic grades and training years (*P*<0.05). The scores in summer sports were lower than that in winter sports (*P*<0.05, 95% CI -0.504–00.042). IA athletes have the highest scores in all athletic grades’ athletes (*P*<0.05, 95% CI 0.030–0.500). Athletes with more than 6 years of training experience had a significantly higher score than those with less than 3 years (*P*<0.05,95%CI 0.80–1.110). Then, we did a statistic for each question (S2 Table in S1 File). Only 31.0% athletes knew that rehydration should be carried out before, during, and after training, which was scarcer among women, lower-athletic grades athletes, or athletes with lower training years (*P*<0.05). Few athletes (1.9%) chose the right dangers of dehydration.

**Table 2:** Mean scores of knowledge and attitude of fluid replacement (Mean ± SD).

Demographic group	Knowledge of fluid replacement	Attitudes of fluid replacement	Knowledge and attitudes of fluid replacement
**Total**	2.8±1.3	2.3±0.6	5.1±1.6
**Sport events**			
*S*	2.7±1.3	2.3±0.7	5.0±1.5
*W*	3.0±1.3	2.4±0.6	5.4±1.6
Statistic	T = -2.322	T = -2.550	T-2.978
*P*-Value	0.021*	0.011*	0.003**
**Gender**			
*M*	2.7±1.2	2.3±0.7	5.0±1.5
*F*	2.8±1.3	2.3±0.6	5.1±1.6
Statistic	T = -1.181	T = -0.849	T = -1.341
*P*-Value	0.238	0.396	0.181
**Athletic grades**			
*IA*	3.0±1.3	2.3±0.7	5.2±1.6
*NA*	2.8±1.3	2.3±0.6	5.1±1.5
*FA*	2.6±1.2^a^	2.3±0.7	4.8±1.5
Statistic	F = 3.856	F = 0.560	F = 2.692
*P*-value	0.022*	0.571	0.069
**Training years**			
*Q*1	2.3±1.2	2.3±0.6	4.7±1.5
*Q*2	2.7±1.1	2.3±0.6	5.0±1.4
*Q*3	3.0±1.3^a^	2.3±0.7	5.3±1.6^a^
*Q*4	2.8±1.3^a^	2.3±0.6	5.1±1.6^a^
*Q*5	2.9±1.3^a^	2.4±0.7	5.2±1.5^a^
Statistic	F = 4.069	F = 0.367	F = 2.700
*P*-value	0.003**	0.833	0.030*

Maximum score: Knowledge: 7, Attitudes: 3.

*Significant level of *P* < 0.05

**Significant level of *P*< 0.01.

^a^ Significant difference with *IA* or *Q*1 of same sport

^b^ Significant difference with *NA* or *Q*2 of same sport.

There were significant differences in the scores of AFR among sport events (*P*<0.05). The scores in summer sports were lower than that in winter scores (*P*<0.05, 95% CI -0.266–60.035). Less than half of the athletes were interested in rehydration knowledge, and the proportion of winter athletes who were interested in rehydration knowledge was higher than that of summer athletes (*P*<0.05, S3 Table in S1 File). In spite of this, most athletes (89.9%) said they would change their fluid replacement habits if it would improve their athletic performance (S3 Table in S1 File).

In the TFR, it was lower in summer sports compared with winter sports (*P*<0.01, 95%CI -0.703–.0.144). And athletes with less than 3 training years had the lowest score (*P*<0.05, 95%CI -1.180–-0.040).

#### Mean scores of KU, AU, and TU

There were differences in the scores of KU (Table 3) in gender and athletic grades (*P*<0.05). The scores of female athletes were higher than those of males *(P*<0.01, 95%CI 0.194–0.653). The total scores increased with an increase in athletic grades. Then, we did a statistic for each question (S4 Table in S1 File). We found that less than half of athletes (42%) knew that light yellow is the normal color of urine, and the number of male was lower than that of female significantly(*P*<0.01). And FA athletes had the lowest proportion(*P*<0.05).The same gender difference also existed in whether rehydration behavior is related to urination behavior.

**Table 3:** Mean scores of knowledge and attitudes of urination (Mean ± SD).

Demographic group	Knowledge of urination	Attitudes of urination	Knowledge and attitudes
**Total**	3.0±1.5	2.1±0.8	5.1±1.8
**Sport events**			
**S**	3.0±1.5	2.1±0.8	5.1±1.8
**W**	3.0±1.4	2.1±0.9	5.1±1.4
Statistic	T = 0.318	T = 0.052	T = 0.285
*P*-Value	0.751	0.959	0.776
**Gender**			
*M*	2.7±1.5	2.2±0.8	5.0±1.9
*F*	3.2±1.4	2.0±0.8	5.2±1.8
Statistic	T = -3.621	T = 2.681	T = -1.703
*P*-Value	<0.001**	0.008**	0.089
**Athletic grades**			
*IA*	3.4±1.6	2.1±0.7	5.5±1.8
*NA*	2.9±1.5^a^	2.1±0.9	5.0±1.8^a^
*FA*	2.9±1.4^a^	2.2±0.9	5.0±1.7^a^
Statistic	F = 6.863	F = 0.408	F = 4.891
*P*-value	0.001**	0.665	0.008**
**Training years**			
*Q*1	2.8±1.5	2.1±0.9	4.9±1.8
*Q*2	3.0±1.5	2.2±0.8	5.1±1.8
*Q*3	3.2±1.4	2.0±0.9	5.2±1.8
*Q*4	2.9±1.4	2.1±0.8	5.0±1.8
*Q*5	3.1±1.6	2.3±0.8	5.3±1.9
Statistic	F = 1.418	F = 2.316	F = 0.859
*P*-value	0.226	0.056	0.488

Maximum score: Knowledge: 6, Attitudes: 4.

*Significant level of *P* < 0.05

**Significant level of *P*< 0.01.

^a^ Significant difference with *IA* or *Q*1 of same sport.

There was a gender difference in the scores of AU (*P*<0.05, 95%CI 0.048–0.310). Male showed a better attitude, although all athletes were less positive. Because only a fifth of athletes were interested in the knowledge of urination (S5 Table in S1 File).

However, for TU, there a was significant difference in athletic grades(*P*<0.05). IA athletes had the highest scores (*P*<0.05, 95%CI 0.120–1.000, 95%CI 0.020–1.000).

#### Correlations between TFR and TU

There was a moderate positive correlation between TFR and TU (r = 0.351, *P*<0.001). The total scores of knowledges and attitudes for fluid replacement expressed 12.3% of the variation of the total scores of knowledges and attitudes on urination.

#### Factors associated with participants’ knowledge and attitudes

The total score of knowledge and attitudes was 10.2±2.8. In the multiple linear stepwise regression model, the participants’ total scores of TFR and TU were used as dependent variables. Gender, sport events, athletic grades, and training years were used as independent variables. The results demonstrated that athletic grades and sport events were the main factors influencing the total scores (Table 4).

**Table 4:** Factors associated with participants’ knowledge and attitudes.

Demographic group	The total scores of knowledge and attitudes			
	**B**	**T**	***P* value**	**95% CI for B for Lower Bound -Upper Bound**
**Athletic grades**	-0.479	-3.028	0.003	-0.789–-0.168
**Sport events**	0.532	2.060	0.040	0.025–1.040

#### The most desirable way of knowledge

**Fig 1:**
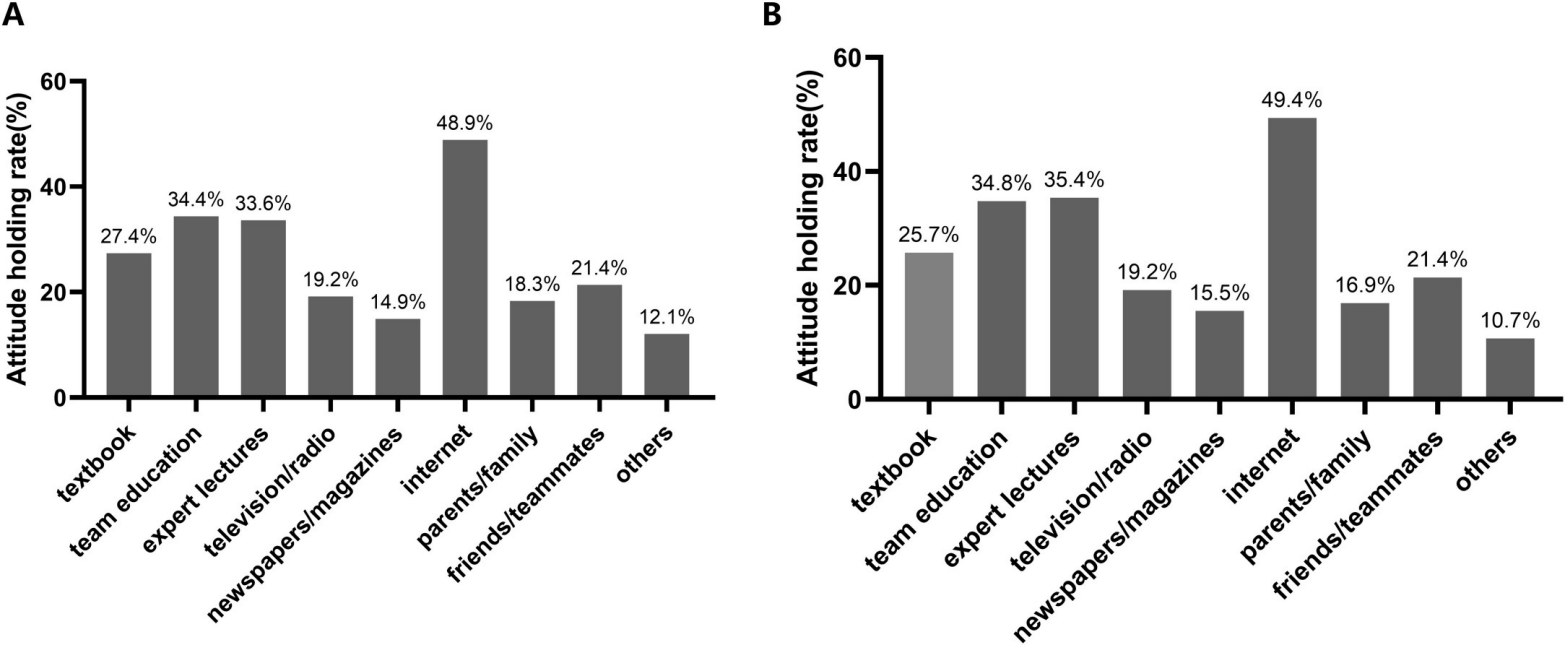
The desirable ways of knowledge. (A) The desirable ways of knowledge on fluid replacement; (B) The desirable ways of knowledge on urination behavior.

The most desirable way for athletes to acquire fluid replacement knowledge was internet (48.9%), followed by team education (34.4%) and expert lectures (33.6%). Internet (49.4%) was also the most popular way for athletes to obtain knowledge of urination, followed by expert lectures (35.4%) and team education (34.8%) (Fig 1).

### Practices among Chinese elite athletes

**Fig 2:**
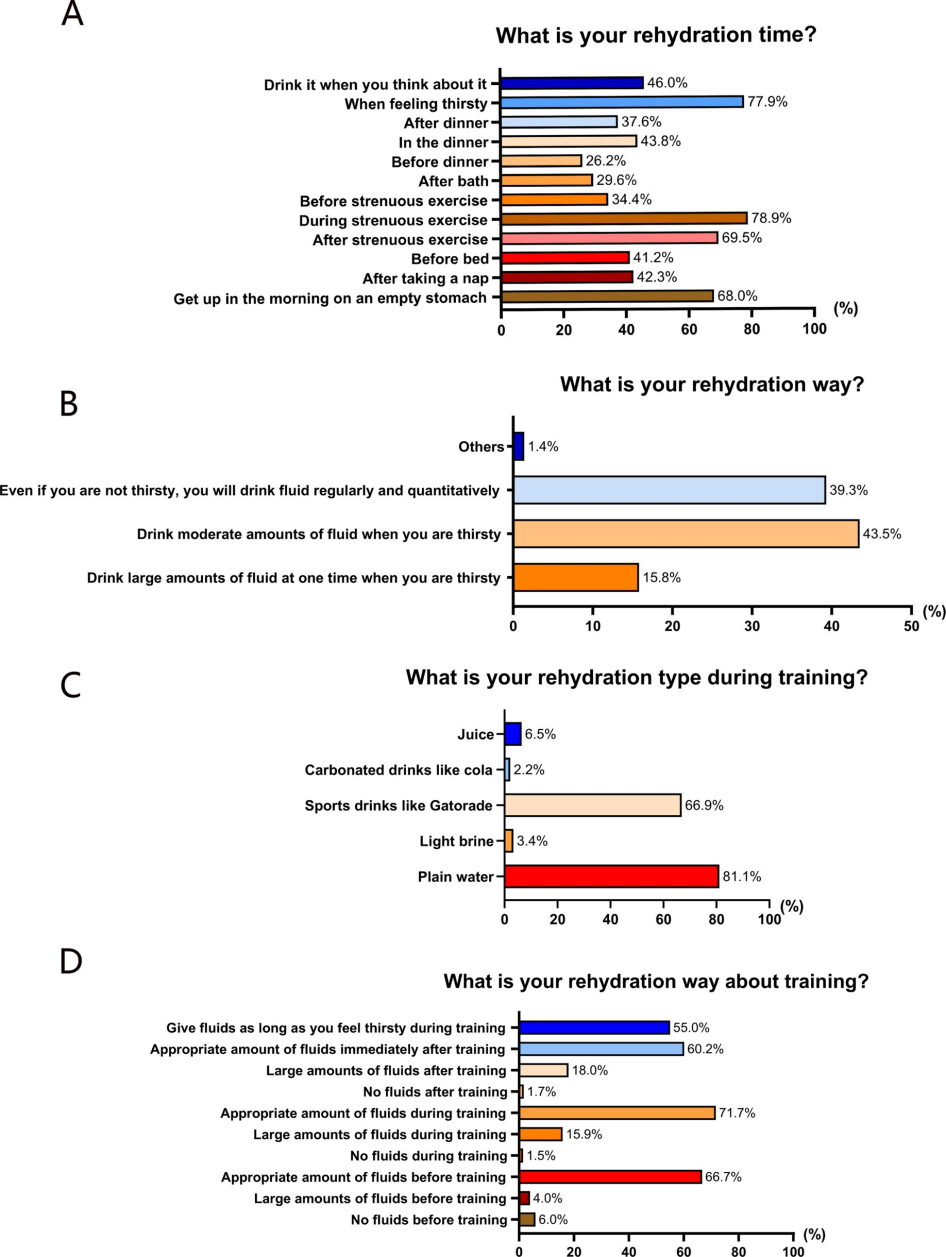
Practices of fluid replacement. (A) Rehydration time; (B)Rehydration way; (C) Rehydration type during training; (D) Rehydration time about training.

We examined the proportion of athletes with fluid replacement behaviors related to rehydration time, rehydration way, and rehydration about training. 77.9% athletes said they rehydrated themselves when they were thirsty. Three-quarters of athletes said they rehydrated themselves during or after strenuous exercise. It showed that only 39.3% athletes took fluid regularly and quantitatively even if they were not thirsty. Plain water is the most popular type of rehydration for training. Two-thirds of the athletes said they took the appropriate amount of fluid before, during or after training (Fig 2).

**Fig 3:**
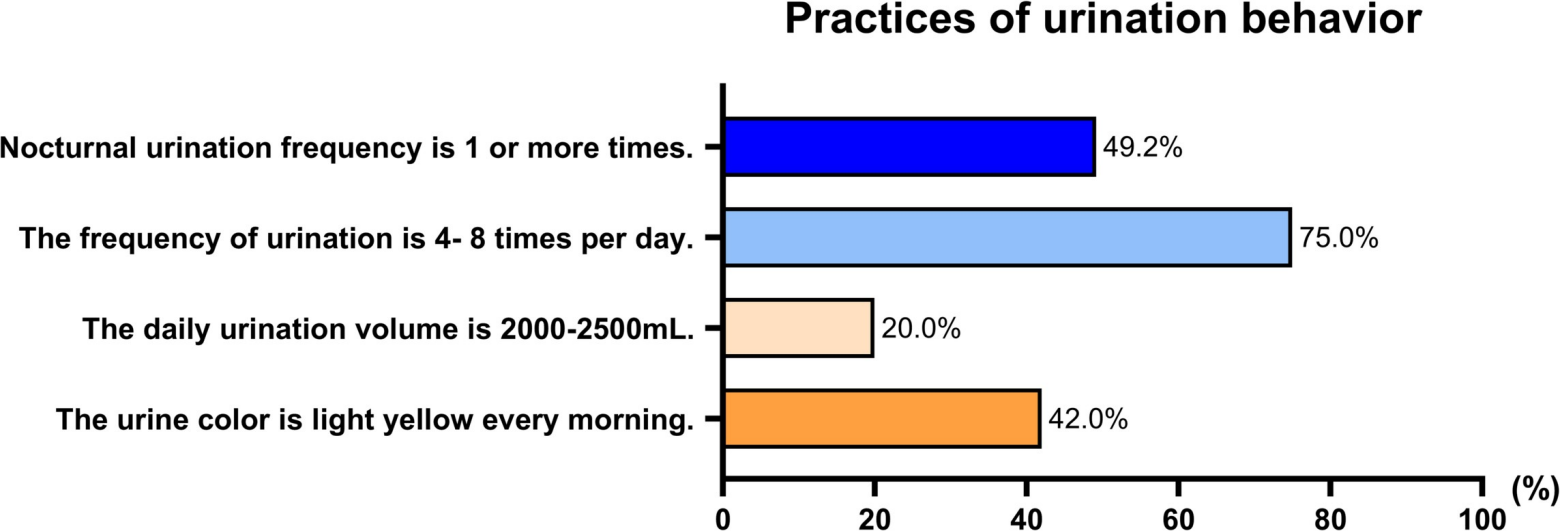
Practices of urination behavior.

We also examined the proportion of athletes about practices of urination behavior. 49.2% had nocturnal urine. Only 20.0% athletes’ daily urine volume was 2000-2500mL. And 42.0% said their urine color was light yellow (Fig 3).

## Discussion

The major findings from this study suggested that: a) High scores of knowledge and attitude on fluid replacement were associated with high urination behaviour scores, and the total scores were mainly related to the sport events and athletic grades. b) For the knowledge and attitude of fluid replacement, the winter athletes did a little better although the scores of all the athletes were not high. Improving their athletic performance was an important reason to attract them to improve their fluid replacement. In terms of practice, thirst is still the main reason for rehydration. c) For the knowledge and attitudes of urination, athletes with higher athletic skill level performed better. Although male athletes scored worse on knowledge than female, they had a better attitude. But when it comes to urination practices, all seem to perform poorly. In general, the results of this study showed that the mastery of hydration knowledge of Chinese elite athletes was very scarce.

There were pieces of literature on general nutrition related to KAP of athletes in which consists of some hydration practice [23,24], but the minimal research evidence about the KAP of fluid replacement, especially for the combination of fluid replacement and urination. Athletes were often dehydrated due to their occupational particularities, so it is crucial to improve their knowledge for better hydration and to correct their hydration attitude because the knowledge and attitude could finally affect their practices. In this case, collecting data on elite athletes could make it easier to tailor their hydration plans for better performance in competition. A survey of American college athletes’ KAP of fluid replacement showed that they had a better acquisition of hydration knowledge [25]. In this study, Chinese elite athletes showed inadequate hydration knowledge, because their knowledge scores were generally lower than 4 points. Although they were better than the ordinary Chinese residents (28.4%) [26] and the Beijing sports population in universities (18.2%) [18] in knowing the correct rate of daily fluid replacement. For example, only 46% of athletes knew that rehydration was needed before exercise. The inadequacy of fluid replacement knowledge weakens the athletes’ interest in rehydration. The results showed that only 41.3% athletes were interested in fluid replacement knowledge, and summer sports athletes had a lower proportion. However, our questionnaire showed that most athletes were willing to learn the knowledge of fluid replacement if their sports performance can be improved. Therefore, the improvement of sports performance can be regarded as the focus of health education.

In terms of practices, thirst was the main reason for rehydration (77.9%) for athletes, which is same as Judge’s results [15]. Dehydration caused by sweating in summer sports athletes was very common, but for winter sports, some events (such as ice hockey [27]) might cause an even higher sweat rate because of their high intensity [28,29]and heavy clothing [30]. At the same time, due to the inhalation of a large amount of cold air during winter sports, which leads to the water and heat loss from lower respiratory tract [31], athletes could have an even more urgent need for rehydration. Therefore, these may be the reasons why athletes in winter sports need more fluids than those in summer sports. Nevertheless, it has to be said that due to the limitation of venues and clothing, we can see that athletes ingest very little fluid during exercise in winter sports (data not shown). The optimization of venues and clothing shall be also considered in the future to facilitate rehydration. What’s more, the number of athletes who rehydrate before, during and after exercise is small, which is similar to that of American college football players [32]. The rehydration practices of Chinese athletes were bad. Sweating can lead to a large amount of electrolyte loss [33]. Athletes often try electrolyte-containing drinks to counteract electrolyte loss caused by sweating during training. Therefore, in our study we observed that many athletes chose sports drinks like Gatorade as a fluid supplement.

Urination is an important option to evaluate the hydration status of athletes. In this study, we find that there is a correlation for the scores of knowledges and attitudes between fluid replacement and urination. It suggests that knowledge about hydration and urination shall be combined during the educational process. Chinese athletes’ knowledge of urination was very limited (3.0±1.5), although female athletes (3.2±1.4) were better than male athletes (2.7±1.5). We have expected that female athletes may have better knowledge of urination because the studies have shown that female elite athletes are more likely to suffer from incontinence and other diseases related to high intensity exercise [34,35]. Athletes were not very positive about urination, because only 18.0% athletes were interested in urination knowledge. And we still need to increase their enthusiasm for urination knowledge. However, 82.0% athletes said that if the cause of abnormal urination were found, they would take the initiative to correct or adjust it. Therefore, team doctors, coaches and researchers are encouraged to help athletes actively discover and correct their urination practices.

Usually, the normal colour of urine is light yellow. The colour of urine has a linear relationship with urine specific gravity and urine osmotic pressure [36]. A study has shown that the accuracy of evaluating hydration status by athletes using urine colour charts is higher than 70% [37]. The results of this study demonstrated that less than half of the urine samples was light yellow, indicating that the athletes were dehydrated. Since the female athletes have a special menstrual period [38], they are more likely to be dehydrated compared to male athletes. A number of studies have also shown that the frequency of urination is associated with the hydration status [39,40]. The normal frequency of urination per day for adults is 4–8 times. The International Continence Society defines nocturia as a frequency of ≥1 instance of waking up at night to urinate [41]. Our results have shown that the frequency of urination in some athletes was not in the normal range, and nearly half of the athletes had nocturia. Oliguria indicates insufficient hydration, while frequent urination and nocturia may suggest the occurrence of cystitis and other diseases [42,43], which will not only affect the health, but also affect the sleep of athletes.

Dehydration affects core body temperature and muscle performance during exercise. When muscle performance declines, muscle glycogen demand increases as fuel. Thus, it decreases sports performance [44]. Dehydrated ultra-endurance cycling participants have greater fatigue and pain than euhydrated participants [45]. It has been reported that only mild hypohydration can decrease cycling performance, possibly by inducing greater thermal and cardiovascular strain [46]. Therefore, hydration education for athletes is urgently needed. It has been confirmed that educational intervention on hydration improves hydration status and enhances exercise performance in athletic youth [47]. Atkins WC et al. [48] have reported that educational intervention improves hydration status and behaviours in high school football players. Martín-Payo [49] has also reported that feasible educational intervention improves hydration behaviour in adolescent soccer players, but hydration guidelines should be based on personal factors. In a word, an appropriate and personalized rehydration education is necessary.

## Limitations and suggestions

It is important to acknowledge the limitations of the current study. There are several factors to consider that may have influenced the results of this study. First of all, it must be admitted that we do not measure with objective tools, so it will be a lot of subjectivity in the data, and the athletes may present themselves in a more positive way, but the actual results may be worse than they are now. Another limitation of this study is that the number of participating athletes in winter sports is relatively small, so the analysis of some categories may not fully represent this group. In a future study, more winter sports athletes should be recruited to get more quantitative data although this kind of questionnaire survey is voluntary. However, the findings of this study confirm the need for education on fluid replacement and urination behaviour among Chinese athletes. Generally speaking, we should conduct more personalized publicity and education based on athletic grades and sport events. Sports nutritionists and coaches, etc. should not only teach athletes about rehydration and urination knowledge, but also provide a favourable environment for the development of their positive attitudes and better practices. Sports teams should use online platforms and expert’s lectures to actively promote the development of athletes’ fluid replacement and urination knowledge and attitude.

## Conclusions

This study demonstrated that, overall, Chinese elite athletes did not have sufficient KAP on fluid replacement and urination. There were differences in some KAP items in athletes of different genders, but their overall performance was poor. Athletic grades and sport events were the associated factor influencing knowledge and attitudes on fluid replacement and urination, athletes with low athletic grades had worse KAP and summer athletes had worse hydration performance than winter athletes. There was a correlation between total scores of knowledges and attitudes for fluid replacement and urination, it means that fluid replacement is as important as urination in future health education. It is necessary to make good use of Internet means to carry out hydration health education. Further investigation using objective tools is still required in the future. It is recommended that education should be provided in the early stages of professional training for athletes.

## Supporting information

S1 File (DOCX)
