# Supplementary material for: A questionnaire study on the knowledge, attitudes, and practices of fluid replacement and urination among Chinese elite athletes
Source: PLoS One. 2022 Oct 12;17(10):e0275685. doi: 10.1371/journal.pone.0275685 (PMC9555643; doi:10.1371/journal.pone.0275685)
Supplement: S1 File — (DOCX) [file pone.0275685.s001.docx]

**Table S1. The steps of multiple liner stepwise regression (n（%）).**

| **Variable definitions** | **The independent variables** | **The assignment** |
| --- | --- | --- |
| Y | The total scores of knowledge and attitudes |  |
| X1 | Sport | 1= “summer sports”  2= “winter sports” |
| X2 | Gender | 1= “male” 2= “female” |
| X3 | Athletic grades | 1= “international-class athlete”  2= “national-class athlete”  3= “first-class athlete” |
| X4 | Training years | 1= “N≤3 ”  2= “3＜N≤ 6 ”  3= “6＜N≤ 9 ”  4= “9＜N≤ 12”  5= “N＞12” |

**Table S2. The knowledge of fluid replacement (n（%）).**

| **Demographic group** | **The daily water intake recommended by the Chinese Nutrition Association for adults is 1500mL-1700mL.** | **The recommended way to drink water is drink regularly according to my activity habits and increase the frequency of drinking.** | **Mineral water and purified water are different.** | **"When I feel thirsty, my body has already been dehydrated." The statement is true.** | **Rehydration should be carried out before, during, and after training.** | **Water replacement and fluid replacement are different.** | **The following phenomena are caused by dehydration:**  **1)increased body temperature, 2)decline in physical strength, 3)heat cramps,4)heat stroke, 5)stroke, 6)coma, 7) coronary heart disease, 8)muscle cramps, 9)increased heart rate** |
| --- | --- | --- | --- | --- | --- | --- | --- |
| **Total** | 230（35.6） | 582（90.1） | 124（19.2） | 479（74.1） | 200（31.0） | 156（24.1） | 12（1.9） |
| **Sports** |  |  |  |  |  |  |  |
| *S* | 169（34.4） | 438（89.2） | 88（17.9） | 355（72.3） | 150（30.5） | 115（23.4） | 8（1.6） |
| *W* | 61（39.4） | 144（92.9） | 36（23.2） | 124（80.0） | 50（32.3） | 41（26.5） | 4（2.6） |
| *χ^2^* | 1.252 | 1.805 | 2.136 | 3.643 | 0.161 | 0.590 | 0.585 |
| *P*-Value | 0.263 | 0.179 | 0.144 | 0.056 | 0.688 | 0.442 | 0.444 |
| **Gender** |  |  |  |  |  |  |  |
| *M* | 104（37.8） | 243（88.4） | 50（18.2） | 194（70.5） | 97（35.3） | 45（16.4） | 7（2.5） |
| *F* | 126（34.0） | 339（91.4） | 74（19.9） | 285（76.8） | 103（27.8） | 111（29.9）^b^ | 5（1.3） |
| *χ^2^* | 1.024 | 1.604 | 0.317 | 3.243 | 4.167 | 15.843 | 1.243 |
| *P*-Value | 0.312 | 0.205 | 0.573 | 0.072 | 0.041* | ＜0.001* | 0.265 |
| **Athletic grades** |  |  |  |  |  |  |  |
| *IA* | 53（38.7） | 128（93.4） | 25（18.2） | 115（83.9） | 38（27.7） | 40（29.2） | 1（0.7） |
| *NA* | 119（36.3） | 297（90.5） | 67（20.4） | 229（69.8）^a^ | 118（36.0） | 83（25.3） | 9（2.7） |
| *FA* | 58（32.0） | 157（86.7） | 32（17.7） | 135（74.5） | 44（24.3）^b^ | 33（18.2） | 2（1.1） |
| *χ^2^* | 1.634 | 4.066 | 0.668 | 10.083 | 8.271 | 5.605 | 2.931 |
| *P*-value | 0.442 | 1.131 | 0.716 | 0.006** | 0.016* | 0.061 | 0.231 |
| **Training years** |  |  |  |  |  |  |  |
| *Q*1 | 26（28.0） | 79（84.9） | 13（14.0） | 72（77.4） | 11（11.8） | 13（14.0） | 2（2.2） |
| *Q*2 | 47（36.4） | 114（88.4） | 21（16.3） | 98（76.0） | 38（29.5）^a^ | 27（20.9） | 3（2.3） |
| *Q*3 | 60（38.7） | 147（94.8） | 33（21.3） | 115（74.2） | 54（34.8）^a^ | 47（30.3）^a^ | 4（2.6） |
| *Q*4 | 62（38.0） | 147（90.2） | 35（21.5） | 110（67.5） | 56（34.4）^a^ | 44（27.0） | 2（1.2） |
| *Q*5 | 35（33.0） | 95（89.6） | 22（20.8） | 84（79.2） | 41（38.7）^a^ | 25（23.6） | 1（0.9） |
| *χ^2^* | 3.793 | 7.127 | 3.489 | 5.955 | 20.988 | 9.945 | 1.485 |
| *P*-value | 0.435 | 0.129 | 0.480 | 0.203 | ＜0.001* | 0.041* | 0.829 |

*Significant level of *P* < 0.05，**Significant level of *P*< 0.01. ^a^ Significant difference with *IA* or *Q*1 of same sport，^b^ Significant difference with *NA* or *Q*2 of same sport.

**Table S3. The attitudes of fluid replacement (n（%）).**

| **Demographic group** | **I think that fluid replacement is important for health.** | **I am interested in fluid replacement knowledge.** | **I am willing to change your fluid replacement habits if it would improve my athletic performance.** |
| --- | --- | --- | --- |
| **Total** | 641（99.2） | 267（41.3） | 581（89.9） |
| **Sports** |  |  |  |
| *S* | 487（99.2） | 191（38.9） | 436（88.8） |
| *W* | 154（99.4） | 76（49.0） | 145（93.5） |
| Statistic | 0.044 | 4.988 | 2.937 |
| *P*-Value | 0.834 | 0.026* | 0.087 |
| **Gender** |  |  |  |
| *M* | 272（98.9） | 115（41.8） | 240（87.3） |
| *F* | 369（99.5） | 152（41.0） | 341（91.9） |
| Statistic | 0.626 | 0.047 | 3.759 |
| *P*-Value | 0.429 | 0.829 | 0.053 |
| **Athletic grades** |  |  |  |
| *IA* | 137（100） | 54（39.4） | 119（85.9） |
| *NA* | 324（98.8） | 137（41.8） | 303（92.4） |
| *FA* | 180（99.4） | 76（42.0） | 159（87.8） |
| Statistic | 2.032 | 0.265 | 4.467 |
| *P*-value | 0.362 | 0.876 | 0.107 |
| **Training years** |  |  |  |
| *Q*1 | 93（100） | 37（39.8） | 86（92.5） |
| *Q*2 | 127（98.4） | 49（38.0） | 119（92.2） |
| *Q*3 | 153（98.7） | 63（40.6） | 139（89.7） |
| *Q*4 | 163（100） | 68（41.7） | 141（86.5） |
| *Q*5 | 105（99.1） | 50（47.2） | 96（90.6） |
| Statistic | 3.587 | 2.218 | 3.604 |
| *P*-value | 0.465 | 0.696 | 0.462 |

*Significant level of *P* < 0.05，**Significant level of *P*< 0.01. ^a^ Significant difference with *IA* or *Q*1 of same sport，^b^ Significant difference with *NA* or *Q*2 of same sport.

**Table S4. The knowledge of urination (n（%）).**

| **Demographic group** | **Light yellow is the color of urine that reflects adequate hydration and good health.** | **50-500mL is the normal urine output volume each time.** | **2000-2500mL is the normal urine output volume in a day.** | **4-8 times are the normal urination frequency for adult one day.** | **Holding back urine is harmful to health.** | **Fluid replacement is related to urine volume or urine color.** |
| --- | --- | --- | --- | --- | --- | --- |
| **Total** | 271（42.0） | 313（48.5） | 129（20.0） | 313（48.5） | 531（82.2） | 369（57.1） |
| **Sports** |  |  |  |  |  |  |
| *S* | 202（41.1） | 248（50.5） | 100（20.4） | 242（49.3） | 403（82.1） | 274（55.8） |
| *W* | 69（44.5） | 65（41.9） | 29（18.7） | 71（45.8） | 128（82.6） | 95（61.3） |
| Statistic | 0.551 | 3.467 | 0.202 | 0.571 | 0.020 | 1.448 |
| *P*-Value | 0.458 | 0.063 | 0.653 | 0.450 | 0.886 | 0.229 |
| **Gender** |  |  |  |  |  |  |
| *M* | 98（35.6） | 136（49.5） | 58（21.1） | 127（46.2） | 203（73.8） | 131（47.6） |
| *F* | 173（46.6） | 177（47.7） | 71（19.1） | 186（50.1） | 328（88.4） | 238（64.2） |
| Statistic | 7.839 | 0.193 | 0.377 | 0.988 | 22.980 | 17.586 |
| *P*-Value | 0.005** | 0.661 | 0.539 | 0.320 | ＜0.001** | ＜0.001** |
| **Athletic grades** |  |  |  |  |  |  |
| *IA* | 70（51.1） | 79（57.7） | 36（26.3） | 80（58.4） | 117（85.4） | 83（60.6） |
| *NA* | 136（41.5） | 153（46.6） | 63（19.2） | 150（45.7）^a^ | 262（79.9） | 180（54.9） |
| *FA* | 65（35.9）^a^ | 81（44.8）^a^ | 30（16.6） | 83（45.9） | 152（64.0） | 106（58.6） |
| Statistic | 7.447 | 6.076 | 4.835 | 6.882 | 2.559 | 1.498 |
| *P*-value | 0.024* | 0.048* | 0.089 | 0.032* | 0.278 | 0.473 |
| **Training years** |  |  |  |  |  |  |
| *Q*1 | 29（31.2） | 38（40.9） | 16（17.2） | 48（51.6） | 77（82.8） | 50（53.8） |
| *Q*2 | 47（36.4） | 58（45.0） | 29（22.5） | 64（49.6） | 104（80.6） | 77（59.7） |
| *Q*3 | 80（51.6）^a^ | 88（56.8） | 26（16.8） | 73（47.1） | 129（83.2） | 98（63.2） |
| *Q*4 | 65（39.9） | 74（45.4） | 30（18.4） | 78（47.9） | 136（83.4） | 99（54.6） |
| *Q*5 | 50（47.2） | 55（1.9） | 28（26.4） | 50（47.2） | 85（80.2） | 55（51.9） |
| Statistic | 13.456 | 8.183 | 4.949 | 0.649 | 0.817 | 4.742 |
| *P*-value | 0.009** | 0.085 | 0.293 | 0.957 | 0.936 | 0.315 |

*Significant level of *P* < 0.05，**Significant level of *P*< 0.01. ^a^ Significant difference with *IA* or *Q*1 of same sport.

**Table S5. The attitudes of urination (n（%）).**

| **Demographic group** | **I think that it is necessary to pay attention to my urine volume and frequency** | **If my urination is abnormal, I will actively seek the cause.** | **If the cause of abnormal urination is found, I will take the initiative to correct or adjust it.** | **I am interested in urination behaviour knowledge** |
| --- | --- | --- | --- | --- |
| **Total** | 399（61.8） | 476（73.7） | 530（82.0） | 116（18.0） |
| **Sports** |  |  |  |  |
| *S* | 295（60.1） | 360（73.3） | 403（82.1） | 82（16.7） |
| *W* | 104（67.1） | 116（74.8） | 127（81.9） | 34（21.9） |
| Statistic | 2.455 | 0.140 | 0.002 | 2.191 |
| *P*-Value | 0.117 | 0.708 | 0.968 | 0.139 |
| **Gender** |  |  |  |  |
| *M* | 145（52.7） | 204（74.2） | 222（80.7） | 55（20.0） |
| *F* | 254（68.5） | 272（73.3） | 308（83.0） | 61（16.4） |
| Statistic | 16.561 | 0.061 | 0.563 | 1.357 |
| *P*-Value | ＜0.001** | 0.805 | 0.453 | 0.244 |
| **Athletic grades** |  |  |  |  |
| *IA* | 53（38.7） | 99（72.3） | 118（86.1） | 22（16.1） |
| *NA* | 127（38.7） | 236（72.0） | 263（80.2） | 60（18.3） |
| *FA* | 67（37.0） | 141（77.9） | 149（82.3） | 34（18.8） |
| Statistic | 0.158 | 2.310 | 2.334 | 0.444 |
| *P*-value | 0.924 | 0.315 | 0.311 | 0.801 |
| **Training years** |  |  |  |  |
| *Q*1 | 36（38.7） | 65（69.9） | 74（79.6） | 19（20.4） |
| *Q*2 | 55（42.6） | 98（76.0） | 108（83.7） | 23（17.8） |
| *Q*3 | 63（40.6） | 106（68.4） | 121（78.1） | 15（9.7） |
| *Q*4 | 56（34.4） | 122（74.8） | 134（82.2） | 35（21.5）^c^ |
| *Q*5 | 37（34.9） | 85（80.2） | 93（87.7） | 24（22.6） |
| Statistic | 2.984 | 5.706 | 4.633 | 10.546 |
| *P*-value | 0.560 | 0.222 | 0.327 | 0.032* |

*Significant level of *P* < 0.05，**Significant level of *P*< 0.01. ^a^ Significant difference with *IA* or *Q*1 of same sport.

**Knowledge, attitudes and practices questionnaire of fluid replacement and urination behavior for elite athletes**

In order to ensure the nutrition of the national team athletes preparing for the 2020 Tokyo Olympics and the 2022 Beijing Winter Olympics from the perspective of reasonable hydration, and to realize the personalized guidance of athletes’ rehydration, it is planned to conduct fluid replacement and urination related knowledge, behaviors and attitudes for athletes.

Purpose:

The questionnaire is used to investigate the status quo of the knowledge, behavior and attitude of the national team players in fluid replacement and urination, and to provide a basis for the subsequent formulation of personalized and precise sports rehydration programs.

Method:

The questionnaire is sent to the athlete through the "WEN-JUN-XING" applet on the mobile phone. Athletes can use their personal mobile phones to answer the questions and complete the online submission.

**Notice:**

**You should answer truthfully according to their personal circumstances. Please think carefully and do not submit quickly! Each of your answers is very important to us. If it is a fill-in-the-blank question, please write it directly on the horizontal line. If it is a choice question, click on the option. If it is not indicated as a multiple-choice question, it is a single-choice question. Thank you!**

**Part 1 Basic Information**

| Q | Question | Variables/ Instruction | Answer |
| --- | --- | --- | --- |
| 1 | Gender | 1）Male 2）Female |  |
| 2 | Age | Write |  |
| 3 | Nationality | 1）Han nationality 2）Zhuang nationality 3）Man nationality 4）Hui nationality 5）_____ |  |
| 4 | Morning hight | Write |  |
| 5 | Morning weight | Write |  |
| 6 | Sports | Write |  |
| 7 | Athletic skill level | 1) “international master sportsmen”  2)“national master sportsmen”  3)“national first-level sportsmen”  4)“national second-level sportsmen” |  |
| 8 | Training years | Write |  |

**Part 2 knowledge, attitudes and practices of fluid replacement**

Knowledge

| 1 | What is the daily water intake recommended by the Chinese Nutrition Association for adults? | 1)<1300mL 2)1300-1500mL  3)1500-1700mL 4)>1700mL |
| --- | --- | --- |
| 2 | What is the recommended way to drink water? | 1) Drink a lot of water at once.  2) Drink only when I am thirsty  3) Drink regularly according to my activity habits and increase the frequency of drinking.  4) I don’t know. |
| 3 | Do you know the difference between mineral water, and purified water? | 1)No, I don’t know.  2)Yes, differences. |
| 4 | "When I feel thirsty, my body has already been dehydrated." Do you think this statement is true? | 1. Ture 2. False 3. I don’t know |
| 5 | Which stage do you think rehydration should be carried out during training?（Multiple choice） | 1. Before training 2. During training 3. After training |
| 6 | Do you know the differences between water replacement and fluid replacement? | 1)No, I don’t know.  2)Yes, differences. |
| 7 | Which of the following phenomena are caused by dehydration?（Multiple choice） | 1) increased body temperature, 2) decline in physical strength, 3) heat cramps,4)heat stroke, 5)stroke, 6)coma, 7) coronary heart disease, 8)muscle cramps, 9)increased heart rate |

Attitude

| 1 | Do you think fluid replacement is important for health? | 1)Not important  2)Important  3)Very important  4)I don't know. |
| --- | --- | --- |
| 2 | Are you interested in fluid replacement knowledge? | 1)I am not interested in it.  2)I am somewhat interested in it.  3)I am interested in it.  4)I am very interested in it. |
| 3 | Would you be willing to change your fluid replacement habits if it would improve your athletic performance? | 1)I am not willing to change it.  2)I am somewhat willing to change it.  3)I am willing to change it.  4)I am very willing to change it. |
| 4 | The most desirable way of knowledge on fluid replacement（Multiple choice） | 1)textbook, 2)team education, 3)expert lectures，4)television/radio,  5)newspaper/magazines,6) internet, 7) parent/family, 8)friends/teammates, 9)others |
| 5 | The acquired knowledge of fluid replacement came from（Multiple choice） | 1)textbook, 2)team education, 3)expert lectures，4)television/radio,  5)newspaper/magazines,6) internet, 7) parent/family, 8)friends/teammates, 9)others |

Practices

| 1 | What is your rehydration time? (Multiple choice) | 1)Get up in the morning on an empty stomach  2)After taking a nap  3)Before bed  4)After strenuous exercise  5)During strenuous exercise  6)Before strenuous exercise  7)After bath  8)Before dinner  9)In the dining  10)After dinner  11)When feeling thirsty  12)Drink it when you think about it |
| --- | --- | --- |
| 2 | What is your rehydration way?(Multiple choice) | 1)Drink large amounts of fluid at one time when you are thirsty  2)Drink moderate amounts of fluid when you are thirsty  3)Even if you are not thirsty, you will drink fluid regularly and quantitatively  4)Others |
| 3 | What is your rehydration type during training? (Multiple choice) | 1. Plain water 2. Light brine 3. Sports drinks like Gatorade 4. Carbonated drinks like cola 5. Juice |
| 4 | What is your rehydration way about training? | 1. No fluids before training 2. Large amounts of fluids before training 3. Appropriate amount of fluids before training 4. No fluids during training 5. Large amounts of fluids during training 6. Appropriate amount of fluids during training 7. No fluids after training 8. Large amounts of fluids after training 9. Appropriate amount of fluids immediately after training 10. Give fluids as long as you feel thirsty during training |

**Part 2 knowledge, attitudes and practices of urination behavior**

Knowledge

| 1 | What do you think should be the color of urine that reflects adequate hydration and good health? | 1. Colorless and transparent 2. Light yellow 3. Yellow 4. Dark yellow 5. Light brown 6. Dark brown 7. I don’t know 8. Other colors, please specify _______ |
| --- | --- | --- |
| 2 | What do you think should be the normal urine output volume each time? | 1)>500mL  2)50-500mL  3)<50mL  4)I don’t know |
| 3 | What do you think should be a normal urine output volume in a day? | 1)<500mL, 2)500-1000mL, 3)1000-2000mL, 4)2000-2500mL, 5)>2500mL, 6)I don’t know |
| 4 | How many times do you think a normal adult urinates a day? | 1)1-4 times, 2)4-8 times  3)>8 times, 4)I don’t know |
| 5 | What do you think is the effect of holding back urine? | 1. Harmful to health 2. Good for health 3. No effect on health 4. Don’t know |
| 6 | Do you think the amount of fluid replacement is related to urine volume or urine color? | 1)No, it is not related to urine volume.  2)Yes, it is related to urine volume.  3)No, it is not related to urine color. 4)Yes, it is related to urine color. |

Attitudes

| 1 | Do you think it is necessary to pay attention to your urine volume and frequency? | 1. Yes 2. No |
| --- | --- | --- |
| 2 | If your urination is abnormal, will you actively seek the cause? | 1. Yes 2. No |
| 3 | If the cause of abnormal urination is found, will you take the initiative to correct or adjust it? | 1. Yes 2. No |
| 4 | Are you interested in urination behaviour knowledge? | 1)I am not interested in it.  2)I am somewhat interested in it.  3)I am interested in it.  4)I am very interested in it. |
| 5 | The most desirable way of knowledge on fluid replacement（Multiple choice） | 1)textbook, 2)team education, 3)expert lectures，4)television/radio,  5)newspaper/magazines,6) internet, 7) parent/family, 8)friends/teammates, 9)others |
| 6 | The acquired knowledge of fluid replacement came from（Multiple choice） | 1) family, 2)friends, 3)coach, 4)teammates ,5)teacher,  6)self-study, 7)Others |

Practices

| 1 | What is the usual color of your morning urine? | 1. Colorless and transparent 2. Light yellow 3. Yellow 4. Dark yellow 5. Light brown 6. Dark brown 7. I don’t know   Other colors |
| --- | --- | --- |
| 2 | In the morning (wake up in the morning to before lunch), you usually urinate about _______ times  In the afternoon (after lunch to dinner), you usually urinate about _______ times  In the evening (dinner to before going to bed), you usually urinate about _______ times  During the night (after going to bed to before waking up), you usually urinate about _______ times | Write |
| 3 | What is your daily urine volume? | 1)<500mL, 2)500-1000mL, 3)1000-2000mL, 4)2000-2500mL, 5)2500mL, 6)never noticed or impossible to estimate |
| 4 | When do you usually urinate? (Multiple choice) | 1)Urinate when you feel like urinating  2)Even if there is no intention to urinate, in order to prevent inconvenient time or place when you want to urinate, you will urinate when there is a toilet.  3)Scheduled urination  4)Never pay attention |
| 5 | Do you urinate during exercise? (Multiple choice) | 1)Yes, I often urinate before exercise  2)Yes, I often feel the urge to urinate during exercise and urinate  3)Yes, I often urinate after exercise  4)No, I don’t usually urinate during exercise |
| 6 | Do you usually hold back urine? | 1)Always　2)often　3)Occasionally　4)Rarely　5)Never |
